# Cephalic phase of insulin secretion in response to a meal is unrelated to family history of type 2 diabetes

**DOI:** 10.1371/journal.pone.0173654

**Published:** 2017-03-13

**Authors:** Björn Eliasson, Araz Rawshani, Mette Axelsen, Ann Hammarstedt, Ulf Smith

**Affiliations:** 1 The Lundberg Laboratory for Diabetes Research, Department of Molecular and Clinical Medicine, Institute of Medicine, Sahlgrenska Academy, University of Gothenburg, Gothenburg, Sweden; 2 Department of Clinical Nutrition, Institute of Medicine; Sahlgrenska Academy, University of Gothenburg, Gothenburg, Sweden; University of Bremen, GERMANY

## Abstract

The pre-absorptive cephalic phase of insulin secretion is elicited during the first ten min of a meal and before glucose levels rise. Its importance for insulin release during the post-absorptive phase has been well documented in animals but its presence or importance in man has become increasingly controversial. We here examined the presence of an early cephalic phase of insulin release in 31 well matched individuals without (n = 15) or with (n = 16) a known family history of type 2 diabetes (first-degree relatives; FDR). We also examined the potential differences in individuals with or without impaired fasting (IFG) and impaired glucose tolerance (IGT). We here demonstrate that a cephalic phase of insulin secretion was present in all individuals examined and without any differences between control persons and FDR or IFG/IGT. However, the overall importance of the cephalic phase is conjectural since it was unrelated to the subsequent post-absorptive insulin release or glucose tolerance. One of the best predictors of the incremental cephalic phase of insulin release was fasting insulin level and, thus, a relation to degree of insulin sensitivity is likely. In conclusion, an early pre-absorptive and cephalic phase of insulin release is robustly present in man. However, we could not document any relation to family history of Type 2 diabetes nor to the post-absorptive phase and, thus, confirm its importance for subsequent degree of insulin release or glucose tolerance.

## Introduction

Insulin secretion is regulated through a complex of different signals elicited by nutrients, incretin hormones such as GLP and GIP as well as through important autonomic stimuli. Animal studies have also shown the importance of the autonomic nervous system for beta cell proliferation and growth in addition to the early anticipatory cephalic phase of insulin secretion [1].

The pre-absorptive cephalic phase of insulin secretion lasts for around 10 min and is initiated by the anticipatory sight, smell and taste of food and further enhanced by chewing and swallowing the food [1–3]. It is largely elicited by vagal stimulation but also includes non-cholinergic mechanisms [1,3]. Previous extensive studies in man have indicated that the cephalic phase is also important for the glucose-stimulated insulin release and, thus, the post-absorptive glucose tolerance [3,4]. Furthermore, this effect was shown to be unrelated to the release of GIP and GLP1 [3]. Although many studies in animals have supported an important role of the autonomic nervous system and the cephalic phase of insulin release [1,5,6], a recent study claimed that the cephalic phase does not exist in man [7]. However, that study was performed quite differently to previous work by the same authors [3] by clamping the “fasting” glucose levels at 6 mM. This was done in order to mimic early prandial conditions and in hope of facilitating a neurally mediated insulin response [7].

We here examined cephalic and post-absorptive insulin secretion in 31 non-obese individuals, including matched individuals with and without a known family history of type 2 diabetes (first-degree family history; FDR). We allowed the individuals to chew and swallow a simple meal in order to mimic the normal food intake procedure. A major aim was to examine if the putative cephalic insulin secretion was different in FDR compared to control subjects based on the concept that it is important for subsequent post-absorptive insulin response and glucose tolerance. Using this conventional approach to measure cephalic insulin secretion, we conclude that an early insulin response, defined as insulin released within 10 min of chewing and swallowing food and before any post-absorptive increase in glucose levels, is present in man. However, we did not find any clear differences in responses between FDR, individuals without a known family history of Type 2 diabetes or with impaired fasting (IFG) or impaired glucose tolerance (IGT). Furthermore, we did not see any relation between the cephalic phase of insulin release and subsequent post-prandial insulin secretion.

## Materials and methods

The regional ethics review board of the University of Gothenburg approved all procedures of this study (from recruitment and informed consent to the meal tests), which was conducted according to the principles of the Helsinki declaration. We recruited 31 healthy non-diabetic and normotensive men free of regular medication via newspaper advertisements. They were between 30–55 years, had BMI 20–34 kg/m^2^ and with or without FDR.

At initial screening visits, verbal and written informed consent to participation was given and inclusion criteria checked (the consent forms were stored). Body mass index was determined as well as body composition (fat mass and fat mass percentage) using DXA (Lunar Prodigy; Scanex, Helsingborg, Sweden). The precision of the DXA scanner has been estimated from repeated measurements on different days in 9 subjects with 2.4% coefficients of variation for body fat.

At a second visit, we performed a standardized meal test. The participants ingested a tasty muffin and drank 15 ml of unsweetened water, tea or coffee within five minutes. The vanilla-caramel muffin with apple contained 1979 KJ/473 kilocalories; 53.7% from carbohydrates, 42.3% from fat and 4% from protein. We drew blood samples at the following time points: -15, -5, 1, 2, 3, 5, 8, 10, 15, 20, 30, 45, 60, 90 and 120 minutes after ingestion. At each time point we determined plasma glucose, serum insulin and serum C-peptide levels, analysed with a photometric method and immunochemistry ECL, respectively, at the accredited Sahlgrenska University hospital central laboratory.

We assessed individual and overall trajectories in glucose, insulin and C-peptide levels as well as the ratio of C-peptide to glucose and insulin to glucose. Given our aim to investigate the presence of a cephalic phase in insulin secretion, we emphasize the current report on the time interval between -5 and 15 minutes. We thus assessed the change at each time point from baseline for each variable. The value at -5 minutes served as baseline value. Changes from baseline and 95% confidence intervals were computed in order to examine when and to what magnitude significant changes occurred between -5 and 15 minutes. We evaluated the trajectories of each variable individually and compared them in order to discern any discordance that might be consistent with a cephalic phase in insulin secretion.

P-values < 0.05 were deemed statistically significant. R statistics software was used for all statistical calculations.

## Results

Baseline characteristics are shown in Table 1. The participants mean age differed slightly between the groups but there were no differences in BMI, fat mass or fasting glucose, insulin or c-peptide levels.

**Table 1:** Baseline characteristics of the participants.

	Overall	CTR	FDR	p
n	31	15	16	
Age (years)	44.4 (8.4)	40.7 (8.5)	47.9 (6.8)	0.014
BMI (kg/m^2^)	26.3 (3.7)	26.9 (4.3)	25.8 (3.0)	0.412
Weight (kg)	85.4 (14.0)	88.6 (16.3)	82.3 (11.1)	0.217
Length (cm)	180 (6)	181 (7)	179 (5)	0.184
Fat mass % DXA	24.2 (8.6)	23.7 (10.4)	24.7 (6.9)	0.735
Fat mass DXA (kg)	21.7 (10.1)	22.4 (12.1)	21.0 (8.0)	0.706
Fat free mass DXA (kg)	64.3 (6.7)	66.8 (6.6)	62.0 (6.1)	0.043
Fat free soft tissue DXA (kg)	60.8 (6.4)	63.3 (6.4)	58.5 (5.7)	0.033
Plasma glucose (mM)	5.1 (0.4)	5.0 (0.3)	5.2 (0.4)	0.134
Serum insulin (pM)	51.0 (21.5)	52.8 (23.2)	49.3 (20.4)	0.655
Serum C peptide (pM)	0.61 (0.18)	0.62 (0.14)	0.60 (0.22)	0.701

Figures are mean values along with standard deviations (SD) in parenthesis. Abbreviations: FDR = individuals with a first-degree relative with diabetes; CTR = control subjects. P values are derived by means of Student’s t-test.

**Fig 1:**
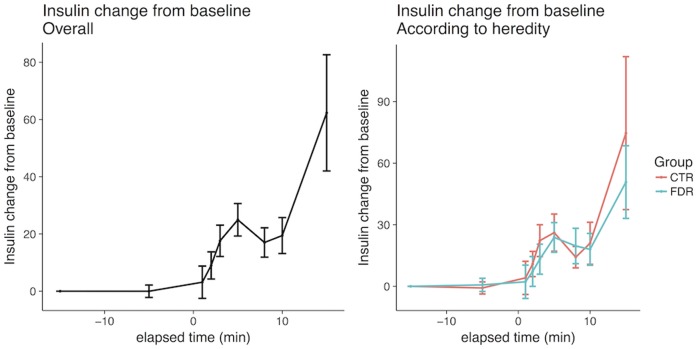
Early cephalic phase of insulin release in all individuals and according to family history of type 2 diabetes. Ctr, control persons; FDR, first degree-relatives of persons with type 2 diabetes.

**Fig 2:**
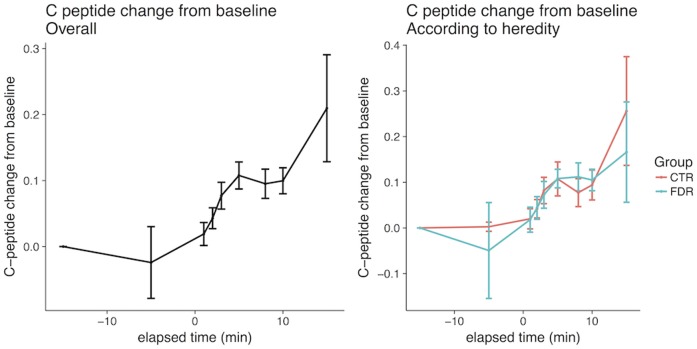
Early cephalic phase of C-peptide release in all individuals and according to family history of type 2 diabetes. Ctr, control persons; FDR, first degree-relatives of persons with type 2 diabetes.

**Fig 3:**
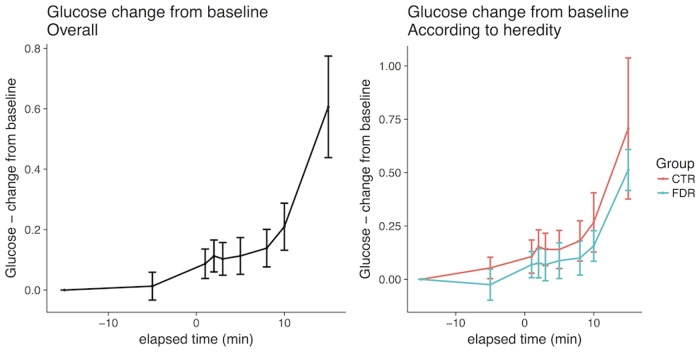
Glucose levels during early cephalic phase in all individuals and according to family history of type 2 diabetes. Ctr, control persons; FDR, first degree-relatives of persons with type 2 diabetes.

As shown in Fig 1-left, there was an early cephalic increase in insulin and, in fact, this was seen in all individuals with a peak after around 3–5 min and this was followed by a marked increase during the post-absorptive period after 10 min. C-peptide levels followed the same pattern, albeit with large inter-individual differences, and increased around 10–15% (Fig 2-left), while the glucose levels remained unchanged and were only significantly increased after 10 min (Fig 3-left). There were no statistically significant changes in insulin, C-peptide or glucose levels between -15 and 0 minutes. There was also no difference in the early AUC for insulin or C-peptide secretion between FDR and healthy control subjects (Table 2, Figs 1 and 2-right), nor in glucose levels (Fig 3-right). Furthermore, there was no significant correlation between AUC for the cephalic phase (0–10 min) and subsequent post-absorptive insulin release or glucose AUC (15–120 min). However, the post-absorptive insulin levels were, as expected, related to fasting levels of glucose and insulin/ C-peptide and, thus, a likely marker insulin sensitivity (Table 2, S1 Fig). Similarly, overall glucose excursions were related to fasting glucose levels (S2 Fig) also within this narrow range of fasting glucose levels.

**Table 2:** Differences in insulin levels (incremental area under the curve) during meal test.

	According to glycemia			According to family history		
	Normoglycemic	IGT/IFG	P value	Controls	FDR	P value
N	27	4		15	16	
iAUC insulin, mean (SD)	18858 (7115)	33205 (13623)	0.002	21648 (11138)	19828 (7428)	0.59
AUC insulin, mean (SD)	25627 (9352)	40610 (15368)	0.010	28847 (13028)	26354 (9453)	0.54
Cephalic iAUC insulin, mean (SD)	119 (79)	74 (53)	0.28	116 (88)	111 (68)	0.88

Figures are mean values and standard deviations (SD). iAUC, incremental area under the curve (1–120 minutes).

**Fig 4:**
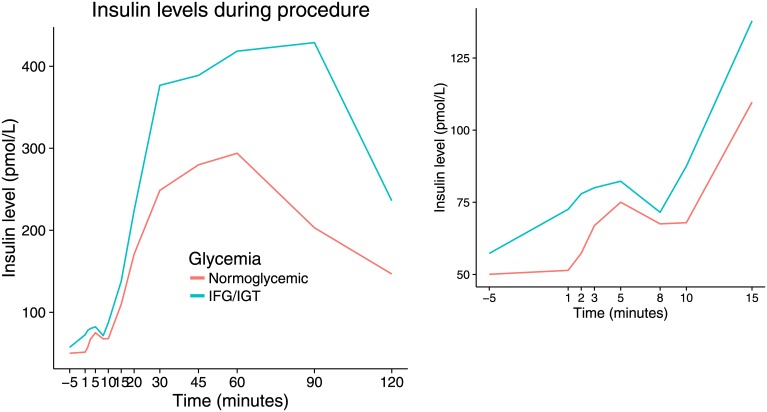
Meal test insulin levels (overall and early phase) in normoglycemic individuals and persons with IFG/IGT. IFG, impaired fasting glucose; IGT, impaired glucose tolerance.

Four individuals were found to have IFG or IGT during the initial OGTT. These individuals had similar cephalic phase of insulin secretion (Fig 4), but significantly enhanced post-absorptive insulin release (Table 2).

## Discussion

Taken together, our results clearly support the presence of an early cephalic and non-glucose mediated insulin response in man. However, the importance of this cephalic phase of insulin secretion for the subsequent glucose tolerance is unclear since we saw no correlations with overall glucose tolerance or insulin secretion. Importantly, neither individuals with a family history of type 2 diabetes or with IFG/IGT exhibited a reduced early response to neural activation. Since we did not measure incretins we cannot exclude differences in their release pattern between the groups. However, it has been repeatedly demonstrated that incretins remain unchanged during the early pre-absorptive phase and, thus, are unrelated to the cephalic phase of insulin release [3,7].

Using a different design to measure the cephalic insulin release, Veedfald et al [7] concluded that a cephalic phase of insulin, glucagon and incretin release did not exist. In contrast, the same authors concluded in a previous paper with different design that the cephalic phase was indeed present and required for a normal postprandial glucose tolerance [3], as also previously suggested in several studies, albeit not consistent in all studies [2,8–10].

The question, then, is why there is inconsistency in results. Most likely is this a consequence of the complex nature of the cephalic insulin secretion rather than differences in the studied cohorts. In our study, like in the previous study by Ahren and Juul Holst [3], a meal was ingested and the individuals were allowed to swallow the food while the study by Veedfald et al [7] used the chewing and spitting procedure together with a clamped fasting glucose level of 6 mM. Clamping the glucose level induced a three-fold increase in the initial insulin levels which may have prevented/attenuated the cephalic phase. In addition, chewing and spitting would also prevent an important contribution of the gastric phase of vagal activation induced by gastric stretching.

Although our data did not specifically compare the outcome of a chew and spit procedure with the full meal ingestion used in our study, we can still conclude that the normal approach in conjunction with a meal is indeed to allow the full neural activation including swallowing. Using this procedure, we see a robust cephalic phase of insulin release but its overall importance for the post-absorptive glucose tolerance is conjectural.

## Supporting information

S1 Fig Relative importance of covariates for predicting insulin levels during meal test. (EPS)

S2 Fig Relative importance of covariates for predicting glucose levels during meal test. (EPS)
